# A Novel Bilayer Wound Dressing Composed of a Dense Polyurethane/Propolis Membrane and a Biodegradable Polycaprolactone/Gelatin Nanofibrous Scaffold

**DOI:** 10.1038/s41598-020-59931-2

**Published:** 2020-02-20

**Authors:** Asghar Eskandarinia, Amirhosein Kefayat, Maria Agheb, Mohammad Rafienia, Moloud Amini Baghbadorani, Sepehr Navid, Karim Ebrahimpour, Darioush Khodabakhshi, Fatemeh Ghahremani

**Affiliations:** 1 Department of Biomaterials, Tissue Engineering and Nanotechnology, School of Advanced Medical Technologies, Isfahan University of Medical Sciences, Isfahan, Iran; 2 Department of Oncology, Cancer Prevention Research Center, Isfahan University of Medical Sciences, Isfahan, Iran; 3 Biosensor Research Center, Isfahan University of Medical Sciences, Isfahan, Iran; 4 Department of Microbiology, School of Medicine, Isfahan University of Medical Sciences, Isfahan, Iran; 5 Department of Environmental Health Engineering, School of Health, Isfahan University of Medical Sciences, Isfahan, Iran; 6 Department of Medical Physics and Radiotherapy, Arak School of Paramedicine, Arak University of Medical Sciences, Arak, Iran

**Keywords:** Preclinical research, Biomedical engineering, Tissues

## Abstract

One-layer wound dressings cannot meet all the clinical needs due to their individual characteristics and shortcomings. Therefore, bilayer wound dressings which are composed of two layers with different properties have gained lots of attention. In the present study, polycaprolactone/gelatin (PCL/Gel) scaffold was electrospun on a dense membrane composed of polyurethane and ethanolic extract of propolis (PU/EEP). The PU/EEP membrane was used as the top layer to protect the wound area from external contamination and dehydration, while the PCL/Gel scaffold was used as the sublayer to facilitate cells’ adhesion and proliferation. The bilayer wound dressing was investigated regarding its microstructure, mechanical properties, surface wettability, anti-bacterial activity, biodegradability, biocompatibility, and its efficacy in the animal wound model and histopathological analyzes. Scanning electron micrographs exhibited uniform morphology and bead-free structure of the PCL/Gel scaffold with average fibers’ diameter of 237.3 ± 65.1 nm. Significant anti-bacterial activity was observed against *Staphylococcal aureus* (5.4 ± 0.3 mm), *Escherichia coli* (1.9 ± 0.4 mm) and *Staphylococcus epidermidis* (1.0 ± 0.2 mm) according to inhibition zone test. The bilayer wound dressing exhibited high hydrophilicity (51.1 ± 4.9°), biodegradability, and biocompatibility. The bilayer wound dressing could significantly accelerate the wound closure and collagen deposition in the Wistar rats’ skin wound model. Taking together, the PU/EEP-PCL/Gel bilayer wound dressing can be a potential candidate for biomedical applications due to remarkable mechanical properties, biocompatibility, antibacterial features, and wound healing activities.

## Introduction

Skin is always at the exposer of different types of damages^1^. Severe skin damages can be life-threatening due to loss of human body fluids, electrolytes, and nutritional components from the wound area. Therefore, wound dressings have gained lots of attention^2^. An ideal wound dressing should protect the wound from external contaminants and facilitate the healing process. However, one-layer wound dressings cannot meet all the clinical needs due to their individual characteristics and shortcomings. Therefore, bilayer wound dressings which are composed of two layers with different properties have gained lots of attention^1,3^. The difference in the structure and characteristics of each layer can provide several advantages^4^. A dense top layer can protect the wound from infection and mechanical stress. In addition, this layer can prevent from wound dehydration and provides a moist environment at the wound area^5^. The sublayer is in direct contact with the wound area and should mimic the structure of extracellular matrix to facilitate cells’ adhesion and accelerate their proliferation^6^.

The utilized materials in a wound dressing can deeply affect its efficacy. Therefore, selection of appropriate materials for the top layer and sublayer synthesis is a determinative step to design an efficient wound dressing. Chitosan, alginate, collagen, gelatin, polyurethane, and polycaprolactone are widely used to prepare different kinds of wound dressings^7–9^. Gelatin (Gel) is one of the most biocompatible and biodegradable polymers which is produced by collagen hydrolysis. Its arginine-glycine-aspartic acid sequence is highly appropriate for cells’ adhesion^10^. Moreover, it contains large amounts of hydroxyproline, glycine, and proline amino acids which potentially accelerate wound healing process^11^. However, natural polymers like gelatin exhibit rapid degradation. Thus, blending of the natural polymers with synthetic polymers can improve the structural stability of the scaffold due to their slow degradation rate. In addition, these blends exhibit better mechanical properties and cell-scaffold interactions in comparison with the scaffolds which are purely composed of natural or synthetic polymers^12^. Polycaprolactone (PCL) is a synthetic polyester characterized by slow degradation rate and high plasticity^13,14^. Recent studies have demonstrated high efficacy of PCL/Gel scaffolds for skin tissue engineering application^15,16^. However, these scaffolds exhibit different weaknesses including poor mechanical properties, unsuitable water vapor transmission rate, and poor anti-bacterial properties. Therefore, utilizing from a protective membrane as the top layer can significantly enhance the efficacy of these scaffolds as wound dressing.

The top layer is response for control of wound microenvironment condition. Moist and incubator-like microenvironment is important to accelerate the healing process and decrease scar formation. A dense membrane can prevent wound dehydration and conserve wound moist. One of the most well-characterized polymers for synthesis of these membranes is polyurethanes (PU)^17,18^. The wound dressings composed of PU have exhibited high efficacy in control of wound moist^19,20^. Also, the top layer should exhibit anti-bacterial properties^21^. Propolis is a natural substance with high efficacy at anti-bacterial and pro-wound healing properties. It is composed of plant exudates, beeswax, and the salivary secretions of bee^22^. Its anti-bacterial properties can be attributed to containing different compounds including ketones, alcohols, steroids, flavonoid, phenolic acids, phenolic aldehyde, and some inorganic compounds^23^. Also, its anti-fungal properties have been demonstrated by previous studies^24,25^. Propolis is a safe and biocompatible natural product with extremely rare reports of allergy incidents. Moreover, propolis can quench and neutralize the free radicals at the wound site^26,27^.

In the present study, PCL/Gel scaffold (sublayer) was electrospun on the PU/EEP membrane (top layer) to produce a bilayer wound dressing. The top layer was a membrane with dense structure and anti-bacterial properties to protect the wound from bacteria and external contaminants. Also, the sublayer was a scaffold with high ability to improve cells’ adhesion and proliferation. The prepared bilayer wound dressing was investigated at morphological, structural, mechanical, antibacterial, and biological properties through *in vitro* and *in vivo* experiments.

## Method and Materials

The utilized propolis was collected from the Shahr-e Kord beehives, Iran. PU (Tecoflex EG-80A) was purchased from Noveon, Inc. (Germany). The applied PU was a medical grade commercial elastomer, an aliphatic poly(ether-urethane) prepared from poly (tetramethylene glycol) (PTMG), HMDI and BDO. PTMG is a polyol polyether with good flexibility and biocompatibility. Tetrahydrofuran (THF) and dimethylformamide (DMF) were purchased from Merck Company (Germany). Gelatin type A (300 Bloom from porcine skin), poly (ε-caprolactone) PCL (MW 80,000), and solvent 2,2,2-trifluoroethanol (TFE) were all purchased from the Sigma-Aldrich (USA). The cell culture materials including RPMI, fetal bovine serum, 0.05% trypsin/EDTA, and phosphate buffer saline (PBS) were purchased from Sigma-Aldrich (USA). Double distilled water was used as the solvent throughout the experiment was purchased from Sigma-Aldrich (USA). L929 fibroblast cell line and Wistar rats were purchased from Pastor Institute of Tehran, Iran. The MTT assay kit was purchased from Sigma-Aldrich (USA). The Staphylococcus aureus (ATCC 25,923), Staphylococcus epidermidis (ATCC 25,925), *Escherichia coli* (ATCC 25,922) and Pseudomonas aeruginosa (ATCC 27,853) were purchased from the Pasteur Institute of Tehran, Iran.

### Preparation of ethanolic extract of propolis (EEP)

Shahr-e Kord suburbs’ beehives were selected for collection of propolis. The ethanolic extract of propolis (EEP) was prepared according to previous our study^28^. The harvested propolis was frozen at −20 for 24 h and then crushed in a blender. 70% ethanol solution was used to dissolve the propolis with a ratio of 1:10 (25 g of propolis in 250 mL of ethanol). The product was kept in a dark incubator at 37 °C for 14 days. After multiple times filtering of the suspension using Whatman No. 4 filter papers, the solvent was removed by employing a rotary evaporator at 40 °C. The product was stored at 4 °C until further uses.

### Preparation of the bilayer wound dressing

**Figure 1:**
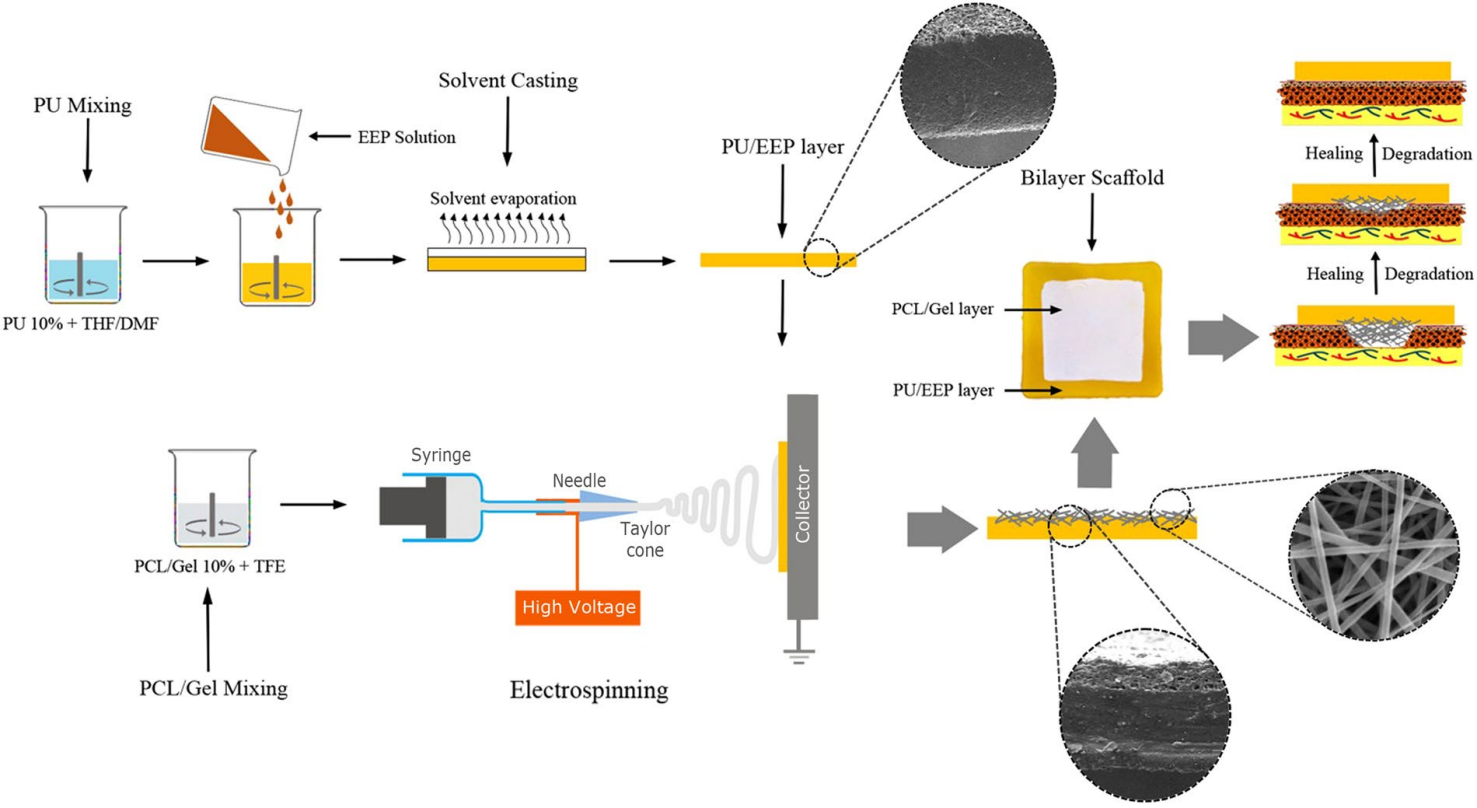
Schematic illustration of the fabrication and preparation of the bilayer wound dressing.

Solvent casting technique was used to prepare the PU/EEP membrane. PU was dissolved into DMF/THF (volume ratio 50:50) and stirred for 3 h at 25 °C to prepare the polymer solution. The concentration of polymer was 10% w/v. Then, EEP was added to the solution for achieving 0.5% w/w concentration and mixed for 1 more hour. The mixed solution was poured in PTFE mold. The cast films were kept at room temperature for 24 h for solvent evaporation. Subsequently, the Gel (10% w/v) and PCL (10% w/v) were separately dissolved in TFE and their solutions were mixed together and stirred at room temperature until achieving a completely transparent solution^29^. The solution consisted of Gel and PCL (50:50) was loaded into a syringe with a metal needle (G22, diameter = 0.41 mm) and then electrospun toward the prepared PU/EEP membranes using an electrospinning machine (Sabz Inc., Tehran, Iran). Figure 1 schematically illustrates the bilayer wound dressing preparation steps.

### Attenuated total reflectance/fourier transform infrared spectroscopy (ATR/FTIR)

The chemical composition of the layers and the possible interactions between their components were investigated by infrared spectroscopy technique. The analysis was performed using an attenuated total reflectance (ATR) cell on the spectrophotometer FTIR-4200 type A (JASCO, USA), in a range of 500–4000 cm^−1^, set on 4 cm^−1^ resolutions and 64 scans.

### Gas chromatography/mass spectrometry

The EEP was analyzed using Gas Chromatography-Mass Spectrophotometry (7890 A, Agilent Technologies, Inc.). About 5 mg of the EEP was mixed with 50 μL of dry pyridine and 75 μL bis(trimethylsilyl) trifluoroacetamide, heated at 80 °C for 20 min and analyzed by GC-MS. Operative conditions were set according to our previous study^30^. The spectrum was analyzed and compounds identified using the NIST05 data library.

### Fiber morphology observation

The morphology and microstructure of the fabricated PCL/Gel scaffolds and PU/EEP-PCL/Gel dressing was observed by employing a scanning electron microscopy (SEM, TESCAN-Vega 3, Czech Republic) at 10 kV accelerating voltage. Prior to performing the analysis, the samples were coated with a thin layer of gold in a sputtering coating device (Q150R-ES, Quorum Technologies, UK). The Image J and MATLAB software were used to calculate the average diameter and porosity of the fibers, respectively.

### Mechanical properties

Instron Universal Testing Machine (Instron Engineering Corporation, USA) was employed to investigated the tensile strength (TS) and elongation at break (E%) of the PCL/Gel scaffolds, PU/EEP membranes, and PCL/Gel-PU/EEP dressings. The PCL/Gel, PU/EEP, and the PU/EEP-PCL/Gel samples were cut using the ASTM standard dumbbell shape template to obtain dumbbell shaped specimens with 30 mm length and 5 mm width. The tensile properties were examined by stretching the samples to break at a crosshead speed of 5 mm/min. The test was repeated five times.

### Hydrolytic and enzymatic degradation

Stability of the PU/EEP and PCL/Gel wound dressings against hydrolytic and enzymatic degradation were investigated according to our previous study^30^. Briefly, the PU/EEP (weight: 0.25284 ± 0.02658 g, diameters: 50 mm × 15 mm, n = 9) and PCL/Gel (weight: 0.03618 ± 0.00334 g, diameters: 50 mm × 15 mm, n = 9) samples were immersed in 10 mL of PBS solution (pH 7.4, 37 °C) for 28 days, to evaluate its stability in an aqueous medium (hydrolytic degradation). The enzymatic degradation behavior of the PU/EEP (weight: 0.25284 ± 0.02658 g, diameters: 50 mm × 15 mm, n = 9) and PCL/Gel (weight: 0.03618 ± 0.00334 g, diameters: 50 mm × 15 mm, n = 9) samples were investigated by the immersion of the samples into a falcon tube containing 10 mL of PBS solution (pH 7.4, 37 °C) with collagenase (0.2 mg/mL) for 28 days. 9 samples were used for each dressing at both hydrolytic and enzymatic degradation conditions (n = 9).

### Contact angle measurement

Water contact angle measurement was used to investigate the wettability of the PU/EEP-PCL/Gel dressing, PCL/Gel scaffold, and PU/EEP membrane. The contact angle meter XCA-50 (USA) was employed to perform the sessile drop technique. Therefore, distilled water was used to place a 4 µL droplet was on the surface of the samples. Triplicate individual measurements were carried out to calculate an average value.

### Propolis release

The propolis release from the PU/EEP membrane was carried according to our previous study^30^. Briefly, the membrane was immersed in PBS under oscillation and the propolis concentration was measured using high-performance liquid chromatography at different time points. The experiment was done in triplicate.

### Anti-bacterial activity

The anti-bacterial activity of the PU/EEP membrane (top layer) was analyzed using inhibition zone (ZOI) test against common wound pathogens. The *Staphylococcus aureus* (ATCC 25923), *Staphylococcus epidermidis* (ATCC 25925), *Escherichia coli* (ATCC 25922), *Pseudomonas aeruginosa* (ATCC 27853), and *Klebsiella pneumoniae* (ATCC 27553) were purchased from Pasteur Institute of Tehran, Iran. Nutrient agar medium was prepared and sterilized according to our previous study^31^. A glass L-rod was used to spread the overnight cultured medium containing bacteria (100 µL) over the nutrient agar medium. The PU/EEP samples were placed over the medium and incubated at standard condition for 24 h. The plates were monitored to measure the clearance zones around the discs. The test was done in triplicate.

### MTT assay

To determine the biocompatibility of the PCL/Gel, PU/EEP and PU/EEP-PCL/Gel dressings with normal fibroblast cells, L929 murine fibroblast cells were purchased from the Pasteur Institute of Tehran, Iran. The PCL/Gel, PU/EEP and PU/EEP-PCL/Gel samples (n = 3) were sterilized by UV light Sirradiation for 30 min and placed at the bottom of 24-well cell culture plates. At least 3 wells were used for each sample (n = 3). Then, home-made Teflon inserts were sterilized by autoclave and used to fix the samples at the wells’ bottom. The Teflon inserts defined a circular seeding area with a diameter of 8 mm. 10^4^ L929 cells were seeded in each well and 400 µL RPMI culture media supplemented with 10% fetal bovine serum (Sigma, USA) and 1% penicillin/streptomycin (Sigma, USA) was added to each well. Also, L929 cells were seeded to 3 wells containing culture medium without any film dressing as the control wells. The plates were incubated in a humidified incubator at 37 °C in a 5% CO_2_ atmosphere and the culture medium was refreshed every 48 h. The cells’ survival was evaluated on the 1^st^, 4^th^, and 7^th^ day of culture using MTT assay according to kit manufacturer (Sigma-Aldrich, USA). The optical density was recorded at 590 nm by a microplate reader (Bio-RAD 680, USA). The test was performed in triplicates.

### Cells and the sublayer interactions

The behavior of L929 fibroblasts on the surface of PCL/Gel scaffold was studied to evaluate the sublayer cytocompatibility. After UV sterilization, the PCL/Gel scaffold was fitted in a 24-well cell culture plate. Each plate was immersed in the culture medium and seeded with 10^4^ L929 cells. Then, the plates were incubated at cell culture incubator for 7 days. Subsequently, the PBS solution was used to remove non-adherent cells through multiple times washing. Then, incubation with 2 vol% glutaraldehyde aqueous solution for 1 h was used for fixing the samples. After multiple times PBS washing, the fixed samples were dehydrated in a graded concentration of ethanol (30, 50, 70, 80, 90, 95, 100%) and dried. The dried samples were immediately sputter-coated with gold and the cells morphology was examined under scanning electron microscope (SEM). This test was repeated three times.

### Animal model and histological evaluation

The animal experiments were done according to our previous studies^30,32^. Briefly, 24 female Wistar rats (6–8 weeks old, 150–180 g) were purchased from Pastor Institute of Tehran, Iran. The rats were maintained at standard condition with complete access to standard rodents’ chow and water. The animals were acclimated for 14 days before entering any experiment. Then, the rats were anesthetized with intraperitoneal injection of Ketamine-Xylazine (Ketamine: 191.25 mg/kg, Xylazine: 4.25 mg/kg) solution. The wounds were created at the rats’ dorsal skin using a punch-biopsy needle with 11 mm diameter after exact shaving and disinfection of the target area. Subsequently, the wounded rats were randomly divided into three groups (n = 8) including control (no-treatment), PU/EEP, and the PU/EEP-PCL/Gel group. The scaffolds were applied precisely on the wound site. The wounds were covered by sterile gauze at the control group. To manage post-operative pain, Ketoprofen (5 mg/kg) was administered subcutaneously until 72 h after operation. The wound healing progression was monitored by continues measurement of the wound diameters using a digital caliper every five days (1^st^, 5^th^, 10^th^, and 15^th^ day). The remaining wound areas’ percentage was calculated based on the below-mentioned Eq. (1). Also, wound’s photographs were captured at the certain days (1^st^, 5^th^, 10^th^, 15^th^ day after operation)^33^.

1 $${\rm{Remaining}}\,{\rm{wound}}\,{\rm{area}}\,{\rm{percentage}}\,=\,\frac{{\rm{wound}}\,{\rm{area}}\,{\rm{day}}\,0\,-\,{\rm{contracted}}\,{\rm{wound}}\,{\rm{area}}\,\text{day}(n)}{{\rm{wound}}\,{\rm{area}}\,{\rm{day}}\,0}\times 100$$

At the 15^th^ day after wound creation, the rats were scarified by overdose of Ketamine-Xylazine mixture (KX). Then, full thickness skin excisions were made from the wound area (n = 8). The obtained specimens were fixed in 10% formalin neutral buffer solution for 24 h. The fixed specimens were processed overnight using an automatic tissue processor (Sakura, Japan). Then, the specimens were embedded in paraffin blocks and a microtome (Leica Biosystems, Germany) was employed to obtain multiple sections with 4 µm thickness. The tissue sections were stained by Hematoxylin & Eosin (H&E)^34^ and Masson’s trichrome^35^ methods, separately. The mounted slides were observed by a digital light microscope which was equipped with an Olympus DP70 digital camera (Olympus, Japan).

### Ethics statement

All animal experiments were carried out in accordance with the Arak University of Medical Sciences’ Guidelines for the Care and Use of Laboratory Animals, which refers to American Association for Laboratory Animals Science and the guidelines laid down by the NIH. All experimental protocols were approved by the ethics committee of Arak University of Medical Sciences (IR.ARAKMU.REC.1398.147). In this study, female Wistar rats were used for animal skin wound model. The minimum required number of animals was used which was enough to obtain reliable results, precise statistical analyzes, prevent from the experiments’ repetition. The rats were maintained at standard condition with complete access to standard rodents’ chow and water. The surgical procedures were done under anesthetize through intraperitoneal injection of Ketamine-Xylazine (KX) solution and at completely aseptic conditions. To manage post-operative pain, Ketoprofen (5 mg/kg) was administered subcutaneously until 72 h after operation. If any signs of post-operative pain, massive necrosis, wound infection or bleeding, failure to eat and drink for over 3 days, inability at limb movement were observed during any steps of the study, the animals were sacrificed by overdose of KX solution. No human subject was used in this study.

### Statistical analysis

The statistical analyses were performed by employing JMP 11.0 software (SAS Institute, Japan) and using one-way analysis of variance (ANOVA) with Tukey’s post-hoc test. The results were displayed as the mean ± standard deviation (SD). The difference was considered statistically significant if P < 0.05. (*P < 0.05)

## Results and Discussion

### Structural properties

**Figure 2:**
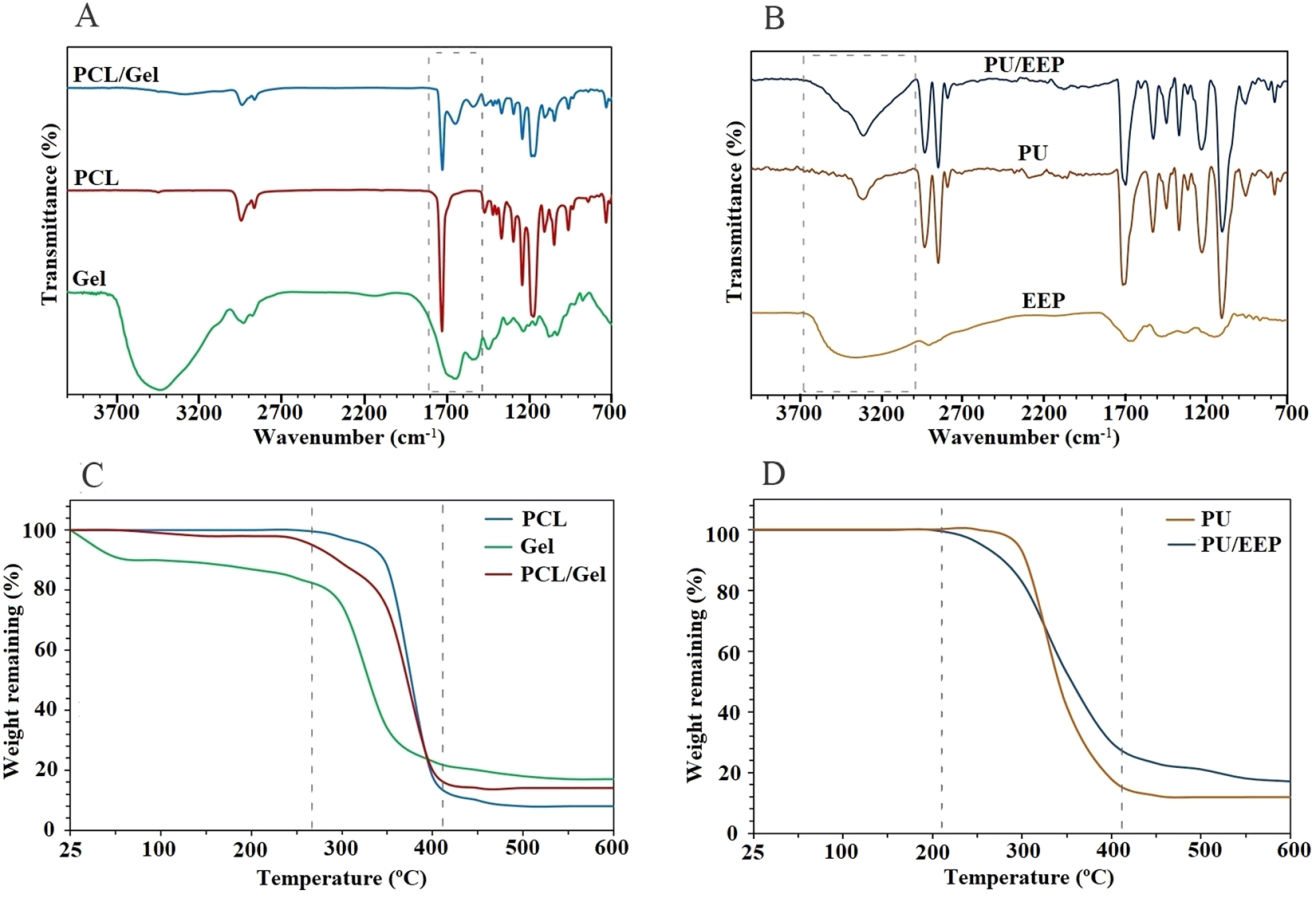
The ATR-FTIR spectra of (**A**) the sublayer and (**B**) the top layer. The TGA graphs of (**C**) the sublayer and (**D**) the top layer.

ATR-FTIR analyses were carried out for characterization of the PCL, Gel, and PCL/Gel scaffolds (Fig. 2A). As Fig. 2A illustrates, the characteristic bands of PCL were appeared at 2949 cm^−1^ (asymmetric CH_2_ stretching), 2865 cm^−1^ (symmetric CH_2_ stretching), 1727 cm^−1^ (carbonyl stretching), 1293 cm^−1^ (C–O and C–C stretching), 1240 cm^−1^ (asymmetric C–O–C stretching), and 1170 cm^−1^ (symmetric C–O–C stretching)^36^. The characteristic bands of Gel were observed at approximately 1650 cm^−1^ (amide I) and 1540 cm^−1^ (amide II) wavelengths. 1540 cm^−1^ peak is related to coupling of N–H bending bond and C–N stretching bond. Also, the 1650 cm^−1^ peak can be attributed to the stretching vibrations of C=O bond which can be observed at both the Gel and PCL/Gel spectra^37^. In addition, the PU, EEP, and PU/EEP spectra are illustrated in Fig. 2B. In the EEP spectrum, stretching vibrations of C-H bonds of the CH_2_ and CH_3_ groups caused formation of high-intensity peaks at 2930 cm^−1^ and 2870 cm^−1^, respectively. The spectroscopy of PU membrane exhibited characteristic absorption bands at 3320, 2960, 1710, 1530, 1220, 1110, and 777 cm^−1^ which represents (N–H), (C–H), and (C–O) bonds on substituted benzene, respectively^38^. The 3000–3700 peak showed the presence of O-H band at EEP. Also, a 3000–3500 cm^−1^ peak at the PU/EEP spectrum was observed which was wider than its counterpart peak at the PU spectrum.

### Thermo-gravimetric analysis (TGA)

TGA was carried out for the PCL, Gel, and PCL/Gel scaffolds to determine changes in weight in relation to change in temperature. The thermal gravimetric curves exhibited a large loss of mass in all samples between 260 and 420 °C, which is related to the characteristic thermal behavior of Gel and PCL (Fig. 2C). The remaining masses were 19%, 8%, and 13% for the Gel, PCL, and PCL/Gel scaffolds, respectively. The PCL/Gel scaffold exhibited remarkable mass loss in the initial decomposition temperature (~ 40–100 °C) in comparison with PCL. Gel has high ability in absorbing moisture. Therefore, this observation can be related to dehydration and loss of the absorbed moisture^39^. Figure 2D shows the TGA curves of the PU and PU/EEP membranes. For the PU sample, the initial decomposition temperature was about 230 °C. As Fig. 2D illustrates, the PU/EEP membrane displayed single-stage thermal degradation. The mass losses were 18% and 11% for the PU and PU/EEP membranes, respectively. The decomposition temperature of the PU/EEP membrane was lower than the PU. This observation can be attributed to incorporation of propolis which has low thermal stability. Moreover, incorporation of EEP to the PU matrix can decrease orientation of the polymer chains’ and crystallinity^40^.

### GC-MS analysis

**Figure 3:**
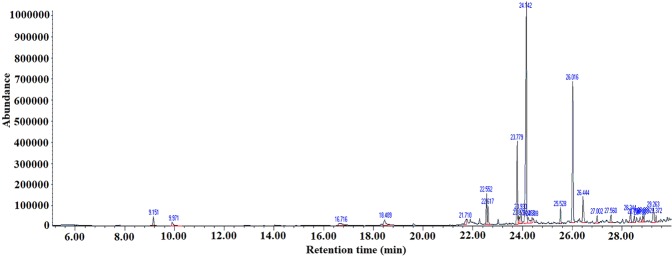
The chromatogram of the EEP according to GC–MS analysis. The figure displays the full-length chart with no cropping.

Propolis is a natural product with promising anti-microbial properties. Its bioactivities are deeply dependent to its chemical composition. In the current study, the chemical composition of the utilized propolis was analyzed using GC-MS (Fig. 3). The identified components belonged to different groups of chemicals. The identified components with significant anti-bacterial properties were illustrated in Table 1. Many aromatic compounds with demonstrated anti-bacterial, anti-fungal, anti-viral, and anti-inflammatory properties, were detected in the utilized propolis^41–43^. The most important phenolic acids and flavonoid derivatives were 1,2-Benzenedicarboxylic acid (2.66%), caffeic acid (1.81%), stearic acid (8.84%), 5,7-Dihydroxy-2-phenyl-4H-1-benzoyran-4-one (32.65%), pinocembrin (2.21%), Icosanoic acid (1.39%), 1-(2,6-dihydroxy-4-methoxyphenyl)-3-phenyl-(1.95%), and naringenin (4.52%). The anti-bacterial activity of propolis can be attributed to the synergistic effect of its various components with significant anti-bacterial properties.

**Table 1:** GC-MS analyzes of the utilized EEP.

RT	IUPAC name	Peak Area (%)	Molecular formula	Structure
22.552	1,2-Benzenedicarboxylic acid	2.66	C_8_H_6_O_4_	
22.617	Caffeic acid	1.81	C_9_H_8_O_4_	
23.779	Stearic acid	8.84	C_18_H_36_O_2_	
23.930	Galangin flavanone (Pinocembrin)	2.21	C_15_H_12_O_4_	
24.142	5,7-Dihydroxy-2-phenyl-4H-1-benzoyran-4-one	32.65	C_15_H_10_O_4_	
24.288	Icosanoic acid	1.39	C_20_H_40_O_2_	
25.528	1-(2,6-dihydroxy-4-methoxyphenyl)-3-phenyl-	1.95	C_16_H_14_O_4_	
26.436	Naringenin	4.52	C_15_H_12_O_5_	

### Morphological observation

**Figure 4:**
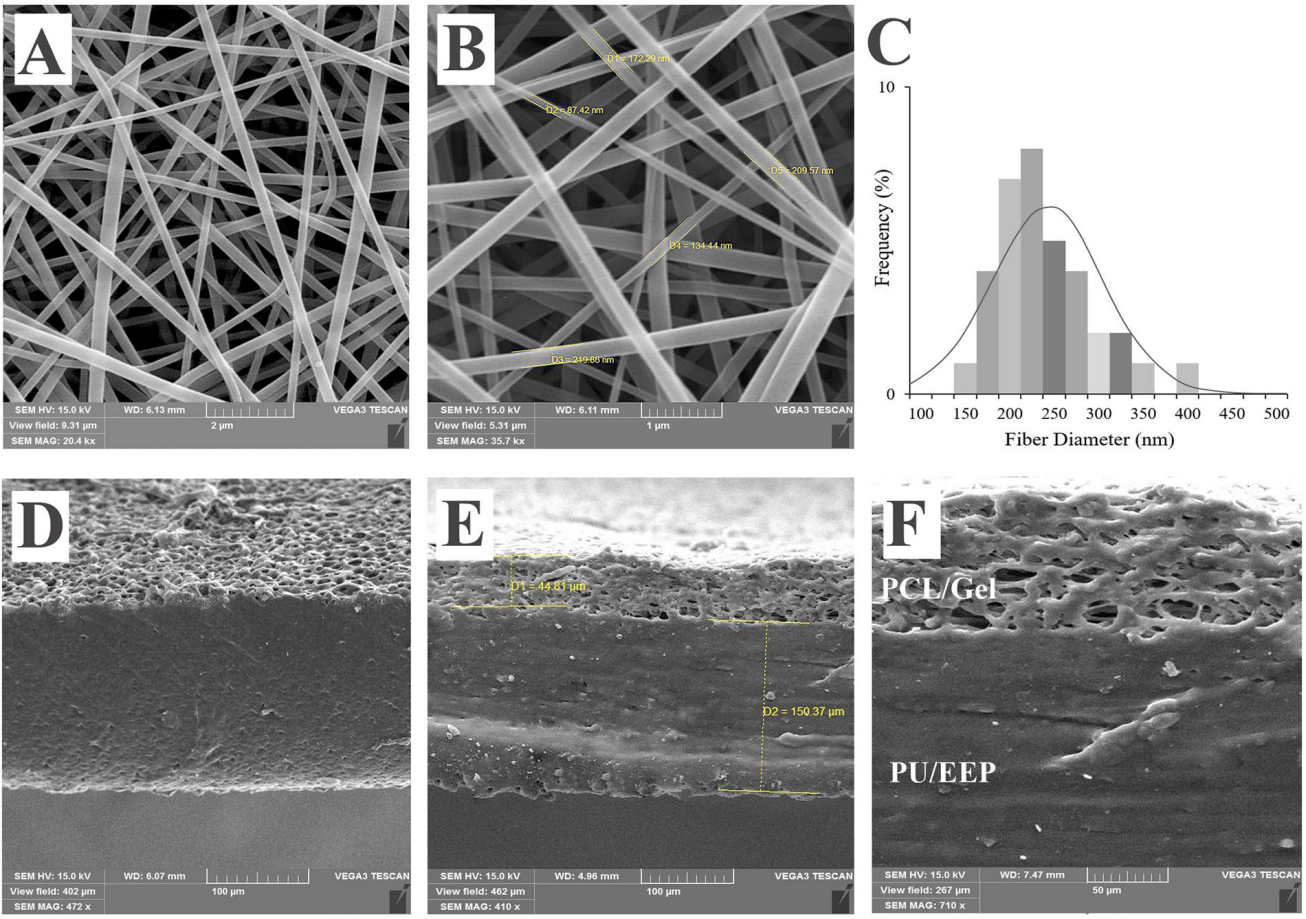
SEM micrographs of the PCL/Gel nanofibers with (**A**) 20400 X and (**B**) 35700 X magnifications. (**C**) The fibers’ diameter distribution chart. The cross-sections of the (**D**) PU/EEP and PU/EEP-PCL/Gel bilayer wound dressing with (**E**) 410X and (**F**) 710 X magnifications.

Surface morphology of the bilayer wound dressing was investigated by SEM (Fig. 4). The SEM images of the PCL/Gel scaffolds under optimized conditions, exhibited a continuous and homogeneous fibrous structure without bead formation (Fig. 4A,B). The average diameter of the PCL/Gel nanofibers was 237.3 ± 65.1 nm, which was calculated from 50 random measurements. The diameters of the PCL/Gel fibers were in the range of 150–400 nm (Fig. 4C). Ramalingam *et al*. reported the fibers’ diameter for the electrospun PCL/Gel hybrid composite nanfabrous woud dressing which ranged from 150 to 250 nm with an average of 234 ± 52 nm^44^. Ajmal *et al*. studied the electrospun PCL/Gel fibers effect on the full thickness wounds and the fabricated PCL/Gel fibers’ mean diameter was 234.1 ± 98.2 nm^45^. In addition, they reported about 74% porosity for the fabricated PCL/Gel dressings. In our study, the porosity was estimated 82.2% which is appropriate for skin tissue engineering applications. Also, average pore size of the PCL/Gel scaffolds was determined 3.2 ± 0.9 µm. Ghasemi-Mobarakeh *et al*. reported no significant difference between mean pores’ size of the electrospun PCL/Gel scaffolds with 50:50 (0.8 ± 0.2 µm) and 70:30 (1.0 ± 0.3 µm) PCL/Gel ratios. Also, mean fibers’ diameter was estimated 113 ± 33 nm and 189 ± 56 nm for PCL/Gel 50:50 and 70:30, respectively^46^. According to previous studies, 60–90% porosity is ideal for scaffolds to facilitate fibroblast cells penetration and proliferation at their structure^47,48^. Also, the porous structure can maintain homeostasis at the wound area by ensuring enough gas and nutrient exchange^49^. The PCL/Gel scaffolds create more space for cells’ migration due to gradual desolvation of the gelatin component. Moreover, appropriate elongation and deformation features of gelatin, facilitate space opening for cells’ penetration in the scaffold structure^50^. At cross-sectional analyses of the wound dressing (Fig. 4D,E,F), the thickness was determined 143.9 ± 6.1 µm for the top layer (PU/EEP membrane) and 52.3 ± 3.4 µm for the sublayer (PCL/Gelatin scaffold). Also, the PU/EEP membrane exhibited a homogenous surface without cracks.

### Mechanical properties

**Figure 5:**
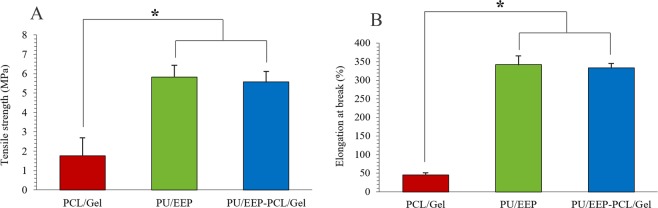
(**A**) The tensile strength and (**B**) elongation at break parameters of the PU/EEP, PCL/Gel, and PU/EEP-PCL/Gel samples.

Mechanical properties of the PU/EEP, PCL/Gel, and PU/EEP-PCL/Gel samples were investigated (Fig. 5A,B). The PCL/Gel scaffold exhibited the elongation at maximum load of 45.9 ± 3.3%, and a tensile strength of 1.7 ± 0.9 MPa. Other studies reported almost the same range of tensile strength like 2.14 MPa^51^ and 1.29 MPa^52^ for the electrospun PCL/Gel scaffolds. Yao *et al*. reported the mechanical properties of PCL/Gel fibers which were electrospun with different blend ratios of 4:1, 2:1, 1:1, 1:2, 1:4. The highest tensile strength and elongation at break values for these scaffolds were 2.9 MPa and ~80%, respectively^53^. As Fig. 5 illustrates, the PU/EEP membrane exhibited significantly higher TS and E% values (TS: 5.8 ± 0.6 MPa, E%: 342.1 ± 23.9%) in comparison with the PCL/Gel scaffold (TS: 1.7 ± 0.9 MPa, E% 45.9 ± 3.3%). On the other hand, the PU/EEP-PCL/Gel bilayer dressing exhibited almost the same values of TS (5.6 ± 0.6 MPa) and E% (333.2 ± 12.4%) in comparison with the PU/EEP membrane (P > 0.05). Therefore, mechanical properties of the PU/EEP-PCL/Gel bilayer dressing can be attributed to the PU/EEP membrane (top layer) and the PCL/Gel scaffold (sublayer) has no significant effect (P > 0.05) on these parameters. Taken together, the PU/EEP-PCL/Gel wound dressing exhibited appropriate mechanical properties due to presence of the PU/EEP membrane as the top layer, which can protect the wound from mechanical damages. Wang *et al*. designed a bilayer wound dressing consisted of collagen-rich extracellular matrix of the porcine small intestine submucosal layer (SIS). This bilayer wound dressing was consisted of a SIS membrane as top layer and a SIS cryogel as sublayer. They reported 35% extensibility for the SIS bilayer wound dressing^54^ which is signficantly lower than the PU/EEP-PCL/Gel bilayer dressing. Thu *et al*. reported alginate based bilayer hydrocolloid films for wound dressing which exhibited ~ 59% extensibility. However, the reported tesnile strength for this bilayer dressing was 27.2 MPa which is signficantly higher than the PU/EEP-PCL/Gel bilayer dressing^55^. However, the ideal tensile strength range for wound dressing and skin cell culture is 0.8–18 MPa^56^.

### Contact angle measurement

**Figure 6:**
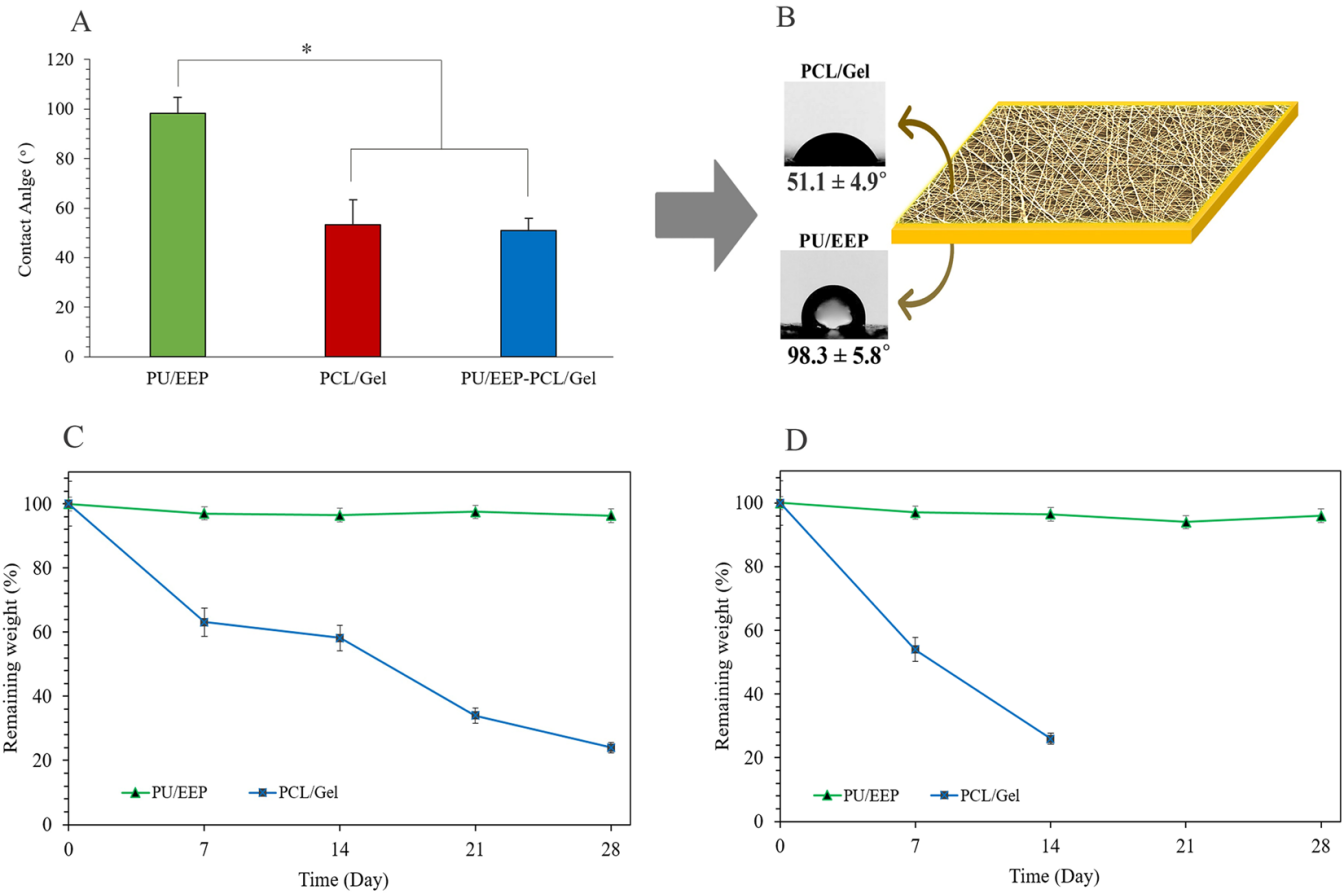
(**A**) The water contact angles of the top layer, the sublayer, and the bilayer wound dressing. (**B**) Schematic illustration of water contact angels of the toplayer and sublayer of the bilayer wound dressing. (**C**) The hydrolytic and (**D**) enzymatic degradation of the PU/EEP membrane (top layer) and the PCL/Gel scaffold (sublayer). Data are expressed as mean ± SD (n = 5).

The water contact angle measurement is a proper technique to measure surfaces’ wettability (Fig. 6A,B). Apropriate hydrophilicity is necessary for the wound dressing’s surface which is in direct contact with the wound area^57^. Therefore, the hydrophilicity of the top layer and sublayer were investigated. The contact angle of the PU/EEP membrane (top layer), PCL/Gel scaffold (sublayer), and the PU/EEP-PCL/Gel bilayer dressing was measured 98.3 ± 5.8°, 51.1 ± 4.9°, and 50.1 ± 2.1°, respectively. As Fig. 6A illustrates, no significant difference was observed between the PCL/Gel scaffold and PU/EEP-PCL/Gel dressing’s contact angels (P > 0.05). The results indicated that the PCL/Gel scaffold has significantly higher hydrophilicity in comparison with the PU/EEP membrane (P < 0.05). This hydrophilicity can be attributed to the multiple hydrophilic functional groups of Gel^58^. Other studies have reported higher hydrophilicity of the electrospun PCL/Gel scaffold (contact angles: ranged ~40–60°) in comparsion with PCL which is moderately hydrophobic (contact angle: ranged ~90–130°)^59–62^. At the study of a PCL/Gel based nanofibers wound dressing by Ajmal *et al*., the PCL contact angel was reported 100.1 ± 3.1° which decreased to 55.5 ± 2.1° after gelatin incorporation at PCL/Gel scaffold. Therefore, the incorporation of gelatin significantly increases the hydrophilicity of the scaffolds because of the amine and carboxyl functional groups in the gelatin structure^63^. The hydrophilic properties of the sublayer can facilitate the cells’ adhesion to the wound dressing’s surface and accelerate wound closure and healing process^64^.

### Hydrolytic and enzymatic degradation

Hydrolytic degradation of the PU/EEP membrane and PCL/Gel scaffold are shown in Fig. 6C. The samples were immersed in PBS for 28 days for evaluation of hydrolytic degradation. The PCL/Gel scaffold lost 36.9%, 58.2%, and 76% of its primary weight until the 7^th^, 14^th^, and 28^th^ day, respectively (Fig. 6C). The PU/EEP membrane exhibited significantly slower degradation rate in comparison with the PCL/Gel. After 28 days, only 1.9% weight loss was observed at the PU/EEP membrane which can be attributed to release of EEP in the PBS and no significant changes were observed at its appearance. The PCL/Gel scaffold exhibited faster degradation rate in comparison with the PU/EEP membrane at hydrolytic degradation test. PCL is a crystalline polymer and Gel is an amorphous polymer in the PCL/Gel scaffold’s structure. The amorphous regions degrade more rapidly than the crystalline regions during hydrolytic degradation of a scaffold^65^.

Enzymatic degradation of the PU/EEP membrane and PCL/Gel scaffold are illustrated as Fig. 6D. The collagenase enzymes are often used for evaluating the enzymatic degradation of wound dressings^66,67^. The PCL/Gel scaffolds which were immersed in the collagenase enzymes solution exhibited 46% and 78% weight loss after 7 and 14 days, respectively. However, the PU/EEP membrane was significantly resistance to both hydrolytic and enzymatic degradation.

### Anti-bacterial activity and propolis release profile

**Figure 7:**
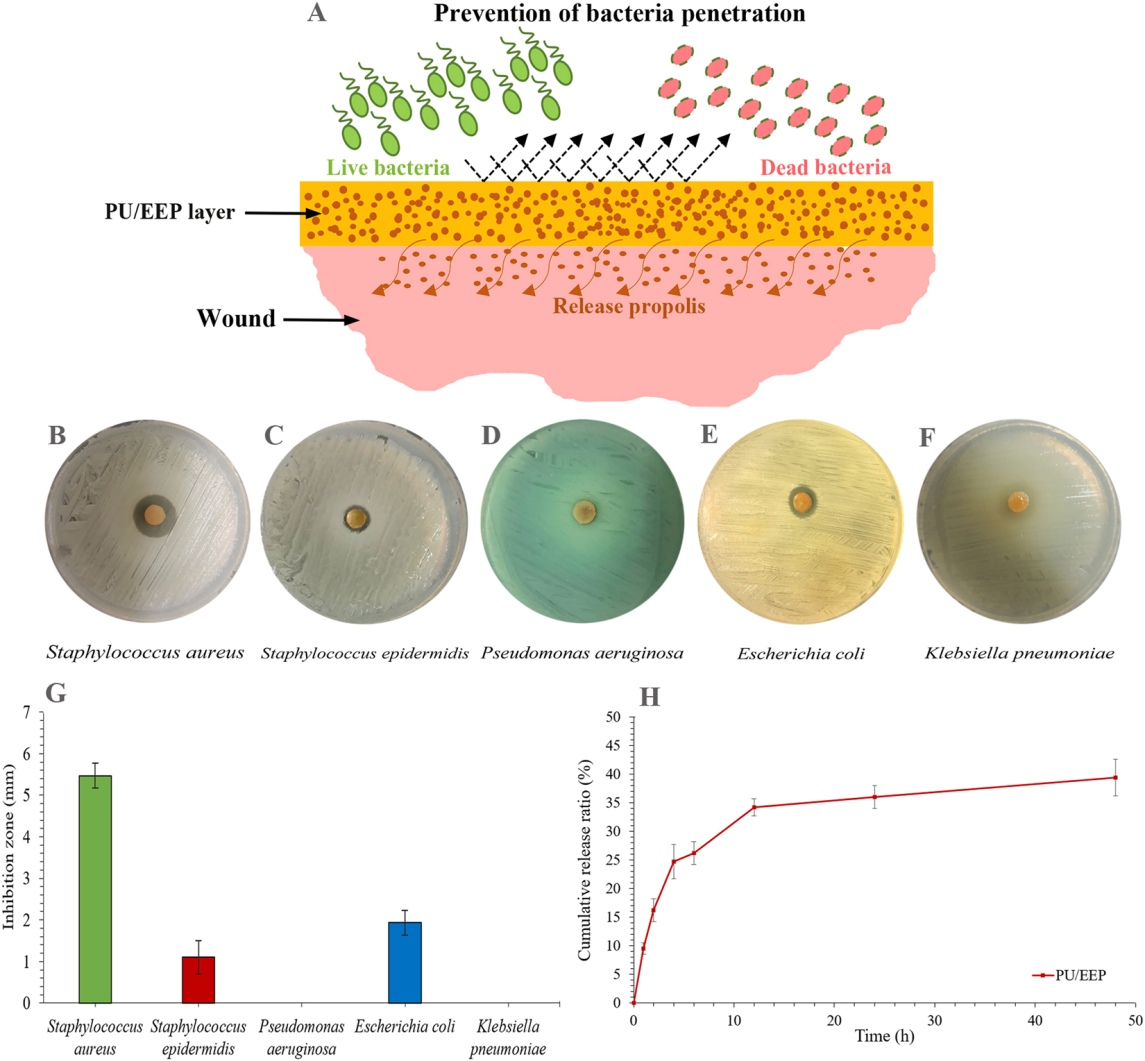
(**A**) Schematic illustration of the prevention of bacteria penetration to the wound area by the PU/EEP membrane. The anti-bacterial activity of the PU/EEP membrane (top layer) against (**B**) *S. epidermis*, (**C**) *S. aureus*, (**D**) *P. aeruginosa*, (**E**) *E. coli*, and (**F**) *K. pneumoniae* according to the inhibition zones method. (**G**) Anti-microbial activity of the PU/EEP membrane (top layer) against different bacteria species according to the inhibition zone method (n = 3). (**H**) The propolis release profile of the PU/EEP membrane (n = 3).

A wound dressing should prevent bacterial penetration and proliferation at the wound area through release of anti-bacterial agents (Fig. 7A). As Fig. 7B, C, D, E, F illustrate, the best anti-bacterial activity of the PU/EEP membrane (top layer) was observed against *Staphylococcal aureus* (5.4 ± 0.3 mm), *Escherichia coli* (1.9 ± 0.4 mm), and *Staphylococcus epidermidis* (1.0 ± 0.2 mm). However, *S. epidermidis* exhibited smaller inhibition zone in comparison with *E. coli* and *S. aureus* (Fig. 7G). This observation can be attributed to the slime layer of this strain which surrounds the bacteria cells and prevents from antibiotics and anti-bacterial agents entrance to the bacteria cells^68^. *P. aeruginosa* was another resistance strain to anti-bacterial effects of the PU/EEP membrane. This resistance can be related to excretion of alginate exopolysaccharide by this stain to perform mucoid layer which can cause the bacteria resistance to anti-bacterial agents^28^. Some studies have incorporated anti-microbial agents into the bilayer wound dressings. Hypericum perforatum oil incorporated bilayer films could exhibit effective anti-microbial activity against *E. coli*, *S. aureus*, and *C. albicans*^69^. Neto, R. J. G. *et al*. reported that chitosan/konjac glucomannan bilayer film can exhibit anti-bacterial activity against both Gram-positive and Gram-negative bacteria^70^. FL *et al*. reported significant anti-bacterial activity by bilayer chitosan wound dressing with sustainable silver sulfadiazine release. The release of sulfadiazine from the bilayer chitosan dressing displayed two phase release including burst release at the first days and then slow release. However, the release of silver from this bilayer wound dressing exhibited a slow release profile. The bilayer wound dressing exhibited high anti-microbial activity and growth inhibition against *P. aeruginosa* and *S. aureus* in agar plates *in vitro* and at infected wound site *in vivo*^71^. Also, Sripriya *et al*. designed a collagen bilayer dressing with ciprofloxacin. This bilayer wound dressing created a 33 ± 3 mm inhibition zone against the mixed culture of *S. aureus* and *P. aeruginosa* and the inhibition zones were maintained for more than 72 h^72^.

The EEP release profile of the PU/EEP membrane was investigated by HPLC after immersing in PBS (Fig. 7H). As Fig. 7H illustrates, two different release phages were detected. The first phage had high slope and continued at first 8 h. This phase is due to the burst release of the loaded propolis on the membrane’s surface. The second phase happened after the initial burst release and exhibited significantly slower release. It continued its upward trend up to 48 h. Therefore, the EEP as the main anti-bacterial agent of the wound dressing is slowly released in the wound area which can cause a long-term anti-bacterial condition at the wound environment.

### Cell viability assay

**Figure 8:**
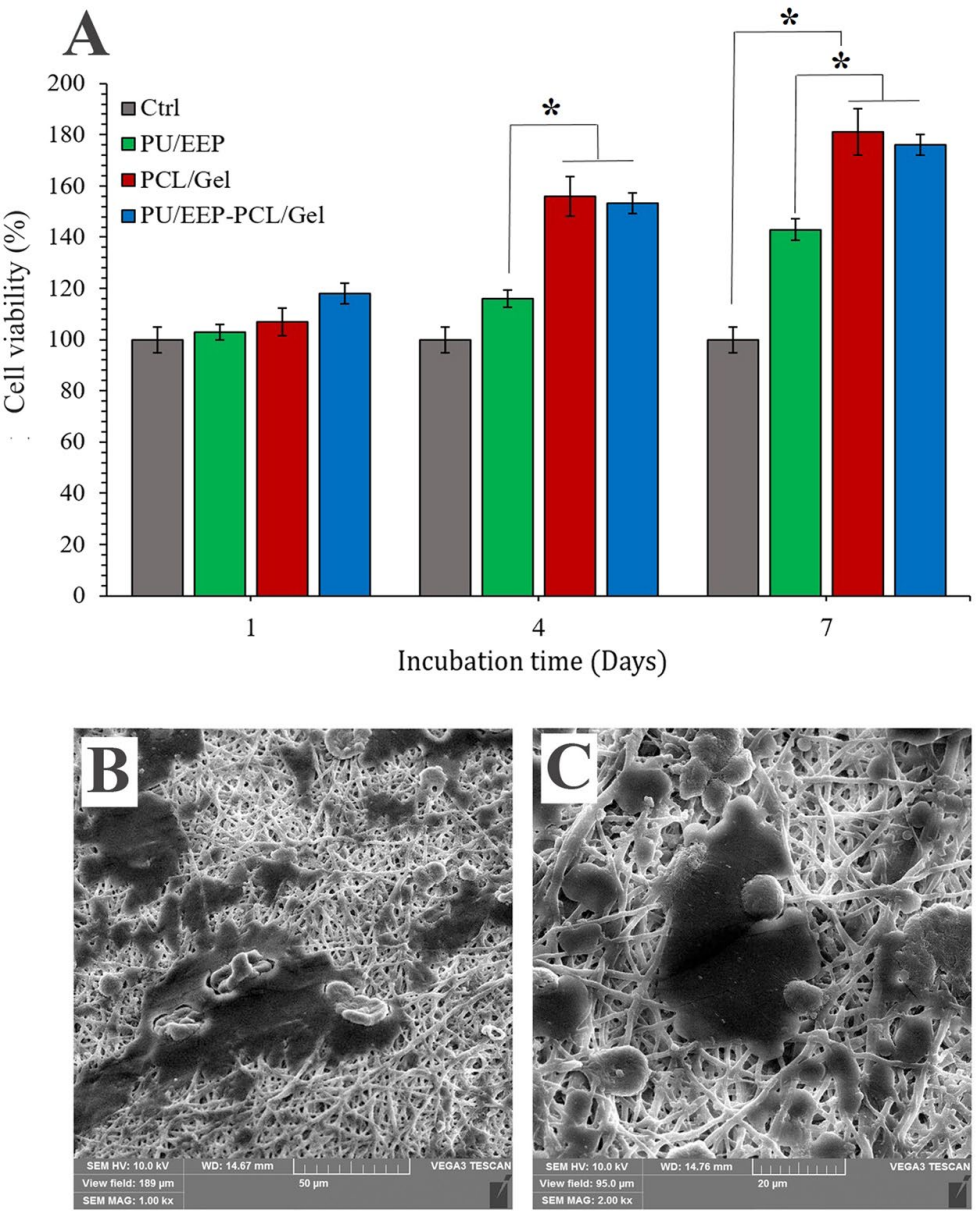
(**A**) Investigation of the PCL/Gel scaffolds’ biocompatibility with the L929 fibroblasts according to cell viability assay after 1, 4, and 7 days incubation (*P < 0.05). (**B**) SEM images of the fibroblast cells on the surface of the PCL/Gel scaffold after 7 days from seeding the cells.

MTT assay was carried out in order to analyze the biocompatibility of the PU/EEP membrane, PCL/Gel scaffold, and the bilayer wound dressing. The cells’ viability was investigated after 1, 4, and 7 days’ incubation with the samples (Fig. 8A). No toxic effect was observed at any of the samples. The PCL/Gel and PCL/Gel-PU/EEP samples exhibited more pro-proliferative effects on the fibroblast cells in comparison with the PU/EEP which became significant from the 4^th^ day of incubation. In our previous study, 0.5 wt% EEP exhibited the most appropriate biocompatibility with L929 fibroblast cells in comparison with 0.25 wt% and 1 wt% concentrations^28^. Therefore, 0.5 wt% EEP concentration was used in this study. Figure 8B,C show SEM images of the fibroblast cells on the surface of the bilayer wound dressing at 7^th^ day. As SEM images exhibit the L929 cells can easily attach and extend on the surface of the PCL/Gel scaffolds. The presence of Gel in the scaffold structure (PCL/Gel) can enhance the cells proliferation. According to previous studies^73^, compositing of a natural polymer like Gel with PCL can improve the hydrophilicity and cellular affinity of the obtained scaffold which provides a favorable environment for cells’ attachment and proliferation.

### *In vivo* wound healing and histopathology analyzes

**Figure 9:**
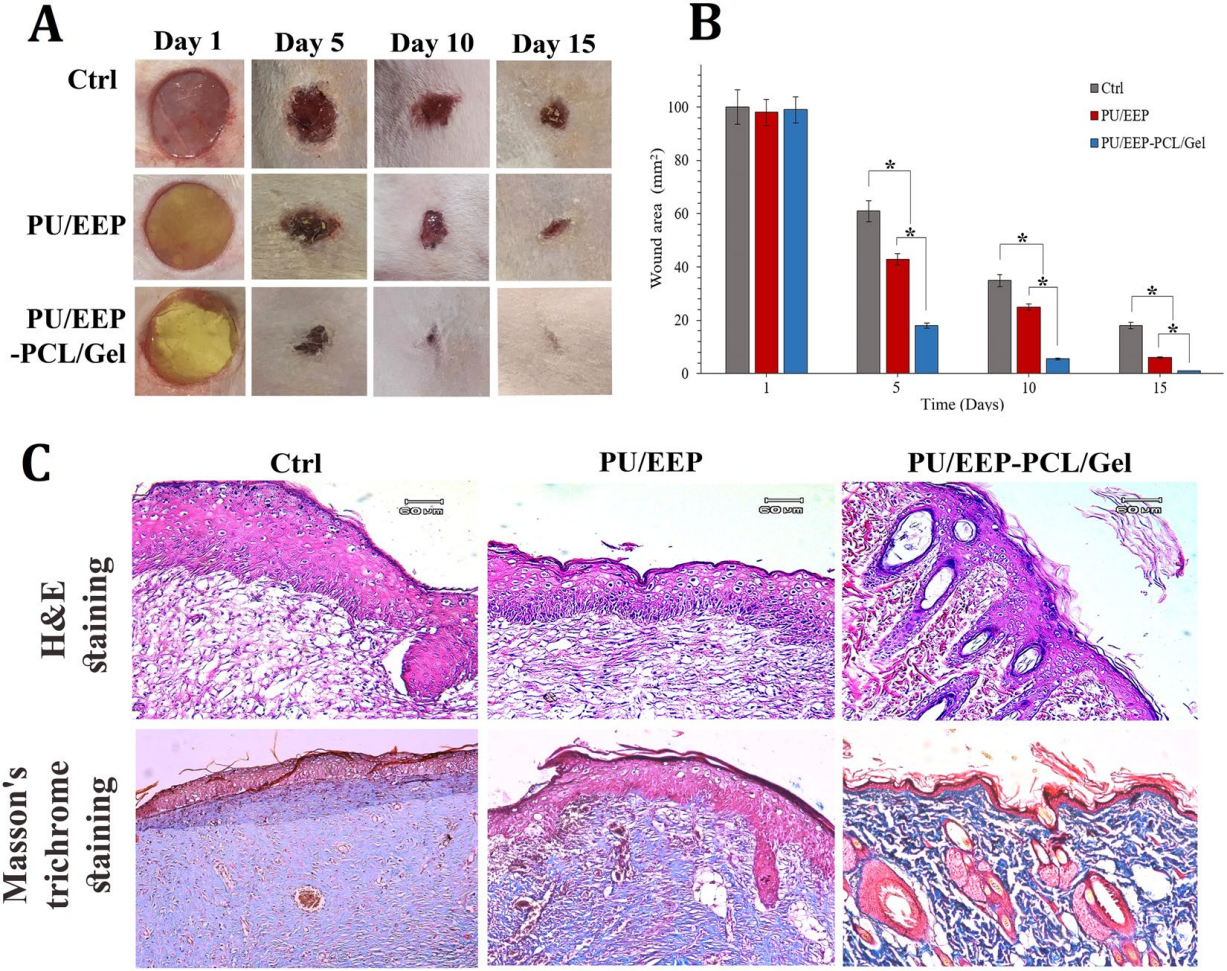
(**A**) Macroscopic photographs of the wounds at definite time points (1^st^, 5^th^, 10^th^ and 15^th^ day after wound creation) to exhibit wound closure progression at different groups (Ctrl, PU/EEP, and PU/EEP-PCL/Gel). (**B**) Wound closure progression according to calculation of the remaining wound area at definite time points (1^st^, 5^th^, 10^th^ and 15^th^ day after wound creation). The data are presented as mean ± SD (n = 8). (**C**) Photographs of the H&E and Masson’s trichrome stained sections of the healed skin specimens at the 15^th^ day after wound creation.

The efficacy of the PU/EEP-PCL/Gel wound dressing was evaluated in comparison with control and PU/EEP group in the Wistar rats’ skin wound model. The PU/EEP and PU/EEP-PCL/Gel dressings were placed at the wound area and the wound closure was monitored for 15 days. All the rats survived throughout the experiment and they were scarified at 15^th^ day for histopathological examinations. The remaining wound area’s percentage was calculated according to wound diameters at the definite time points (1, 5, 10, and 15 days after operation). Figure 9A illustrates the macroscopic photographs of wounds at control, PU/EEP, and PU/EEP-PCL/Gel groups during time progression. The wounds at the control group were just covered by gauze. On the 15^th^ day, the PU/EEP group exhibited higher wound closure rate in comparison with the control which can be mainly attributed to maintaining the moist environment at the wound site^74^. In addition, the PU/EEP-PCL/Gel wound dressing treated group exhibited approximately healed and closed wounds at this day. As Fig. 9B shows, the remaining wound area of the control group was 100%, 60.3%, 36.3%, and 17.9% at the 1^st^, 5^th^, 10^th^, and 15^th^ day, respectively. Also, the remaining wound area of the PU/EEP treated wounds at the 1^st^, 5^th^, 10^th^, and 15^th^ day were 98.2%, 42.1%, 25. 4%, and 8.5%, respectively. The remaining wound area percentages of the PU/EEP-PCL/Gel treated group at the same time points were 99.0%, 20.1%, 7.9%, and 2.6%, respectively. These observations demonstrate the PU/EEP-PCL/Gel potential to accelerate wound healing process. Figure 9C illustrates histopathological evaluation of skin specimens from the healed wound area at 15^th^ day after wound creation. The PU/EEP-PCL/Gel group specimens exhibited significantly more developed dermis in comparison with the PU/EEP and Ctrl specimens due to presence of lower number of inflammatory cells and development of more hair follicles^75,76^. Also, the specimens were stained with Masson’s trichrome to analysis collagen deposition. Collagen deposition at the wound matrix plays a critical role in the healing process, as it provides scaffolds for wound-healing cells^35^. More accumulation of collagen fibers and collagen deposition was observed in the PU/EEP-PCL/Gel group’s specimens in comparison with the PU/EEP membrane and Ctrl. Moreover, more densely packed collagen fibers with a parallel arrangement was observed in the extracellular matrix of the PU/EEP-PCL/Gel group’s specimens in comparison with other groups. These observations can demonstrate appropriate wound healing activity of the PU/EEP-PCL/Gel wound dressings according to histopathological analyzes. Yao *et al*. investigated keratin-gelatin composite bilayer dressing *in vivo*. They created full-thickness rectangular wounds (1.5 cm × 1.5 cm) on the back of adult male Sprague–Dawley rats. The reported bilayer wound dressing caused complete wound closure at 14^th^ day post-surgery. They reported accelerated dermis development and early formation of hair follicle and sebaceous glands at the bilayer wound dressing groups^77^. Xu *et al*. investigated their microporous silicone rubber membrane bilayer in BLAB/c mouse wound model. Full-thickness defects (10 mm × 10 mm) were created on the back of the mice and monitored for 7 days. It was observed that on the 7^th^ day, the mean wound remaining area of the control, vaseline gauze, and bilayer wound dressing groups were 70%, 62.8%, and 35.8%, respectively. However, the amount of wound closure was the same at 1^st^ and 3^rd^ days^78^. Thu *et al*. used alginate-based bilayer hydrocolloid films for treatment of Sprague-Dawley rats’ skin wound model. The full-thickness skin excision wounds were created by a punch-biopsy needle (6 mm diameter and about 1 mm depth) and a 1 cm^2^ area film dressings was applied on the wound. Although the normal saline group exhibited 20% mean wound remaining area after 10 days, the bilayer dressing treated wounds were attained full closure and significant re-epithelialization^55^.

## Conclusions

In this study, PCL/Gel solution was successfully electrospun into a continuous, uniform, and bead-free nanofibrous scaffold over a PU/EEP membrane to form a bilayer wound dressing. This wound dressing exhibited appropriate biocompatibility, biodegradability, and mechanical properties. Also, remarkable anti-bacterial activity against common wound infection bacteria was observed due to presence of the top layer (PU/EEP). In addition, animal studies revealed that the PU/EEP-PCL/Gel bilayer wound dressing can significantly accelerate the wound healing progression and shorten wound closure time. Taking together, the PU/EEP-PCL/Gel bilayer wound dressing can be a potential candidate for biomedical application due to the high biocompatibility and significant anti-bacterial and wound healing activities.
